# Symptomatic Pathology of the Mouth

**Published:** 1885-04

**Authors:** Elton R. Smilie

**Affiliations:** San Francisco, Cal.


ARTICLE II.

### SYMPTOMATIC PATHOLOGY OF THE MOUTH.

BY ELTON R. SMILIE, M. D., SAN FRANCISCO, CAL.

(Continued from last Number.)

After four hours had past in almost total abstraction, from thoughtful self-control or mindful of being led like a child in leading strings, she rose to depart without the slightest allusion to the nature or seat of her disease, and proffered me an unheard of fee, even as a recompense for successful capital operations in surgery which jeopardized life. With the rapidity of startled thought I scanned in review our conversation to see if any portion of it could be constructed into any of the legendary traits of Finnish and Yankee expressions of selfish longing, and although I failed to detect either in feeling or language the motive that has earned for them the stigma of petti-fogging tricks for gain, I could not restrain my tears, impressed with the thought that I had in some way given occassion for her to entertain so poor an opinion of me as to suppose I would take a fee for receiving rather than conferring a service.

Her well-trained perception quickly appreciated the nature of my reflections, and to soothe the wound and make me understand the distinction upon which her apparently prodigal estimation of service rendered had been based, she explained that if the aid had depended solely upon mechanical talent of filling teeth united with the *noli ne tangere* delicacy of touch required in dealing with the nerves to insure a comparatively painless operation, she would have been satisfied with paying me the accustomed equivalent for occupying my time in preparatory consultation. But her requirements were of a higher order than the mere mechanical dentist could fulfill, "of this," she said, "I consider myself competent to judge; for in premonition of the necessity from physicial warnings brought on by the irregularities of my habits, I passed two courses, disguised as a student, in the University of Paris; my studies confirmed me in the belief that I had incurred certain predispositions even after I had relieved my mind from the imaginary fears that leads almost every student to adopt the different maladies described in lectures and books. Acting upon the impulse of impressions I consulted in freedom from disguise all the different professors, and each examined me with all the appliances of his specialty, but no two agreed in stating the uature, or location of the disease, or treatment of my system for the amelioration or cure of the diseased tendency, and were only infallible in the exorbitant fees charged as an evidence of their ability. So you see, child, that without flattery, I have placed a just estimate upon the service you have already rendered me, as the demonstrator of certain symtomatic effects upon parts of the mouth as an evidence of diseased translation from remote organs, —which I have drawn from you unconsciously,—is a true interpretation of my own feelings, and has realized my premonitions, and to-morrow I will give you the opportunity for directly verifying your relative diagnosis. You will perceive from this statement in train for other confirmations, without computing in payment of indebtedness the enlistment of gratitude for corrective reproof of my conscious delinquencies, that I have not been over-generous in money compensation. You have probably heard much that is true of me by common report, and greatly to my disadvantage, but as it is but reasonable to suppose that persons have a better knowledge of their own acts and the motives that prompted them, to-morrow I will make known to you everything that can possibly have a bearing upon my disease and complications, necessary for your aid in forming a correct diagnosis. But it will be with shame and regretful grief; for you will then realize that even with your manifest inclination, we cannot enjoy the privileges of open friendship, as the reputation of being more sinful than the actual passages of my life, would blast the integrity of yours, allowing that your pitying disposition could overcome its natural abhorrence of instincts which prompted my transgressions, and holding it reasonable that my sufferings from physical retribution would prove a sufficient punishment without the addition of your scorn. You will perceive by these hints that the opinion of the public, usually ungenerous, has done me no great wrong in pronouncing judgment upon the course of my past life. Still you will urge in my defence, that there was a leading period in my life which was blameless of the intention to do wrong, and that example was the incentive that gave me the bias to adopt evil temptations before my perceptions were sufficiently matured to forsee the inevitable results. In this respect your palliative reflection would be just, and my regrets for the influence, and indignation against the personal example of the school teachers who led me astray, are constant, and I have ever retained an emulous admiration for a woman who has preserved her purity unimpeached and conformed to the inborn promptings which her modest nature should recognize as legitimate with the instincts of our sex. These innate impressions confirm me in the belief, that if they had not been misdirected, my natural inclinations were good, and with aid, cultivation would have afforded me in development as true a sorce of happiness as the opposite course has obtained for me an unenviable notoriety. Receive the fee, child, as an evidence of my confidence in your ability to render me both physical and moral benefits that will exceed the power of money to purchase, and if you can overcome your repugnance to the sacrifices of womanly modesty I have made to vanity for the gratification it would afford to hold intellectual supremacy, you will enable me to counteract in a measure the evil influence that I have hitherto exercised in giving freedom to unchaste thoughts in a class of society, that from position controls and shapes the fashion of speech, clothes, and style of movement to all the lower grades of humanity."

As she was persistently earnest in her intention to endow me before trial, she accepted my proposal that it should be recorded in her note book as a proffered recompense that I would accept if my treatment proved beneficial. In parting for the night she made a quick motion, as if to receive and return my proffered kiss, then withdrew her head with salutation, and with a sad expression of face shook my hands and departed.

A sleeples night was passed in reviewing our conversation, and although I could not fail to understand the motive of the evening visit, it had been so skillfully disguised with the interest enlisted and delicate tact, that she accomplished her purpose with a success in developing my latent thoughts that in review greatly surprised me, as unaided by suggestion they would have remained in embroy. But instead of feeling mortified that I had been made a passive instrument of demonstration, I was gratified that I had acquitted myself so well as to gain the confidence of one so gifted in all the arts that contribute to gain the possessor a well-founded reputation for the variations of wisdom and wit, which unfortunately with our sex is usually combined with characteristic traits of folly engendered by attrition in acquisition. In vain I tried to conjecture the nature of her disease, and although she had at times hesitated and placed a check upon her tongue when a certain class of diseases were discussed, I was loath to even think it possible that a person of her refined cultivation could by any vagary of instinctive passion, knowingly expose herself to the chance of contagion from so vile a pollution, and censured myself with injustice for thoughtful preparation in view of the method of treatment should it prove to be a metastatic translation of the kind, and earnestly hoped that my admiring reverence would not be so easily humiliated.

Fortunately the day following was exceedingly cold, so that I felt sure that I should be obliged to accept the proviso of a Russian winter engagement, "I will surely come if it is not *very* cold," for those in the ordinary routine of mechanical operations. As I anticipated, hers was the only engagement kept, and with a sympathetic consideration for the comfort of her servants for which I was prepared to give her credit, she dismissed them with an appointment, and in reciprocal bearing on their part they showed a differential attachment quite opposite in expression to the indifference manifested by my patrons of the higher nobility for the welfare of their servants.

Upon receiving and helping her to disrobe I could not fail to discover from her care-worn face that she had passed not only a sleepless but an anxious night, and when seated in my operating chair her eyelids quivered and it was only with a manifest effort of will that she regained sufficient composure to enable her to give me a relation of the symptomatic cause of her apprehensions. As she proceeded my fears and sympathies were strongly excited, and when it came to an examination of her mouth I found to my dismay its condition the exact counterpart of Mrs. P's, whom you attended during the closing year of my novitiate. The left inferior maxillary wisdom tooth had in it a large gold filling condensed to a perfect weld, that had in the main received an unscratched finish, but a notch here and there of the holding rim showed that with all his care, the dentist had not directed the point of his instrument securely upon the border line of gold necessary for forming a compact junction with the bevel angle wall beneath the rim. Upon inquiry, my patient said the filling of the tooth had caused her intense pain although the pulp was not exposed, but the jar of the instrument upon the dentine seemed to compress it and send a pang with each stroke to the eye and ear, and from the irritation a chronic sensitiveness succeeded, and continued until six months after the operation she took a cold that resolved it into an active state of ulceration that required a month's active counter-irritation to subdue. The second molar had a small filling inserted at the fissure junction in the crown's central depression. With the exception of these, the balance of her thirty-two teeth were in excellent condition, and of that peculiar structure that gives sure indication that in hereditary transmissions the constituents of her bony system had remained free from the taint of degeneration through a long reach of progenitors. This, with the history she gave me of her ancestors' habits, afforded me the hopeful feeling of certainty that I had not the septic infliction of predisposition to contend with. But it was enough for direct diagnosis, that I discovered the liver-colored and shaped fungus starting in growth from the neck attachment of the gum of the diseased wisdom tooth, and extending backward toward the fauces. Fully impressed with the danger I should have to contend with, after the extraction of the tooth from the rapid growth of the fungus, if as with Mrs. P's the seat and cause of translation was uterine irritation during the process of gestation, with a feeling of blushing hesitation I questioned her, as from public report and her personal relation I had received no hint of her being a married woman. With more marked confusion than she had yet shown, after many stammering attempts to speak, she faltered, "Yes, child, it is as you judge, but hoping there would be no necessity, I could not reveal to you so great a cause for shame; but now I might as well make a clean conscience of it, for one's sins are sure to find them out." She then related that she had been five times in the process of motherhood, three of which matured with the birth of two boys separately, and two girls, twins, all free—up to the present time—from developed hereditary disease of any kind; and to secure them from the temptations which had led her astray, and unacknowledged birth paternity, had placed them as the children of a widowed mother, of rank, in the charge of English Episcopal clergymen to receive their education. "Thus you will see, child, mis-steps of the kind lead to a chain of errors which in turn fetter you to a life of falsehood, from which there is no honorable way of escape, except by death, which if slow brings the tormenting fears of reflection that your children may suffer shame on your account." In answer to my inquiry, with regard to the condition of her appetite during the period of gestation, and whether she suffered pathognomonic symptoms, out of the healthy course. She stated that with her first two children she had a normal appetite that ranged within the usual bounds of nutrition, with only an increase adapted to the duplicate process for sustenance; but when enciente the fourth time she took abortives to relieve herself from the incumbrance, and then for the first time experienced the pains of toothache and the vagaries of a morbid appetite. Apparently observing the shock of disappointment that flushed my face from a confession so repulsive to the innate modesty and humane tenderness excited in anticipation of approaching motherhood, she answered to the expression, "Yes, child; *the one* fatal mis-step once taken in girlhood, disrobes the unguarded victim of every charm that should raise her above, and protect her from the brutish instincts of our body's animal propensities; and the more elevated our capacity to realize all that is beautiful in the present life from the cultivation of the ideal, which becomes to the pure woman the echo of immortality, the greater the power she possesses for evil and self-debasement, and alas, that I feel constrained to acknowledge it, to you, I have often exulted in my ability to lead the aping, common herd to their own destruction. But relentless remorse ever haunts me to raise hopes of redemption only to crush them with the extraction of some new form of impetuous passion that leaves me to sink deeper in admiration, but by full confession must startle into self-defence the womanly pride of your innocence; still I am grateful to your emulous mercy that is fully interested for the cure of my self-inflicted physical ills.

Try and bear with me, without feeling yourself humilitated in sex alliance with one who has committed so many gross errors, and now feels acutely that the mental and physical punishment is justly merited, and may be the means through your instrumentality—if my life is spared—of restoring me to a career of usefulness that for a short period of repentance I once enjoyed; and instead of accounting me a murderess, think in extenuation, if you can, of the misery, worse than death, that would have been entailed upon them if born to a sullied and illegitimate heritage."

Despite your cynical sneer at her abortive repentance, and pleading skill to enlist all my mental energies in her behalf, I know, that secretly, you are as soft and sympathetic in your emotions as any woman can be, and would very much quicker have fallen upon her neck and greeted her pleading resolution with tears than I did—perhaps to its mutual undoing—for her power over men in mental accord must have been irresistible, notwithstanding your warning that the physician's mind should be kept free from all compassionate bias while balancing the symptoms with the personal experience of the patient, to obtain a correct diagnosis and indictations for treatment. But after our emotions had somewhat subsided, the necessity of following your advice became strongly impressed upon the patient as well as myself, for the sensational scene had demonstrated the almost insuperable difficulty that sensitively intelligent women will be obliged to overcome before they can command the disinterested control of their feelings in a sufficient degree for the unbiased exercise of perception required for tracing the tendency of disease in all of its variations from cause to effect, necessary for a correct diagnosis and successful treatment. Besides, with the doctress and patient who are affinitised in sentiment the conversation in examination will be too diffusive for speedy decision in complicated diseases, and those of an acute form requiring quick and determined action, of which, you will say, I am giving you ample illustration. Now, I will try and show you that I have taken a lesson from the womanly failings I have acknowledged as the besetting sins of our sex, by giving as concisely as possible the result of my treatment.

Although the disease, in its fungoid development around the wisdom tooth, had made its appearance but three days before, premonitory lancinating pains had been felt in the jaw for a month or more, reaching from the second molar backward to the angle of the jaw. These symptomatic precursors so well defined the location and limits of the inceptive stage of the disease, I was forwarned, and reminded of your experience of the necessity of prompt action, and made my preparations accordingly. But with all my anticipations and forebodings, was not prepared for the rapid development of its malignant character, which succeeded the extraction of the second molar and wisdom tooth; and now wonder that I preserved my self-reliance that enabled me to cope successfully with the aggravated irruption of this formidable disease, for in less than an hour its fungus filled and over-lapped the sockets margins notwithstanding the free use of the powerful caustic with which you supplied me; and but for the instrument you devised for its protection in application would have baffled all my efforts, as a perfect deluge of saliva poured forth from the glands that would have neutralized its effect. Again, fortunately the wash of the saliva prevented the swallowing and absorption of the dissolved poison of which the caustic is composed, for, although I applied it constantly for five hours there was no deleterious effect, other than slight nausea, and then I had the extreme relief of seeing the liver hue turn to white, showing that the mushroom growth of the fungus was stayed. Still, I did not trust to favorable appearances, but continued the application at intervals during twenty-four hours, when the reaction of sloughing commenced and afforded me the first view of the sockets bony margin, and enabled me to probe and penetrate the cellular tissue of the jaw without the root enclosure, which was broken down, and the matter of its decomposition imparted to the instrument the most unbearable odor that ever came within the scope of my nostrils, and produces a repetition of the sensation of disgust whenever I think of it. It surprised me that no blood flowed from the extraction of the teeth, as you had cautioned me to have all my appliances ready to stop it, as it would prove a hindrance to the effective use of caustic, from coagulation. That, there was no flow bood, I ascribed to the pressure of pent-up matter upon the venous vessels; and by allowing the caustic to act more readily, fortunately produced a quicker and more favorable result, that relieved my anixety from a tension almost insupportable.

As a caution against its eruption after the cavity was free from matter, I syringed it thoroughly with a solution of muriate of mercury, using a curved spoon excavator to remove the partially decomposed substance, and cotton fastened to the end of a copper wire sufficiently flexible to clean it more perfectly in every part that there might be no absorption for the secondary production of fragilitas cancrum. This treatment was alternated with the local application of antimonial potash, and administration of the hypophosphites of lime and soda, with variations suited to the symptomatic indications. In my investigation for pathognomonic cause and resulting variations, never was doctor or doctress more favored than I with the intelligent interpretation of symptoms afforded by my patient; but with a sympathetic confidence that encouraged in me self-reliance —the inspired source of ability—she never even suggested a remedial measure; but after convalescence, acknowledged that her curiosity was strongly excited, in every instance, to learn the method my judgment proposed to adopt to combat unfavorable tendencies, and when announced felt a calm assurance of its success. In questioning the source of this reliance, so contrary to your experience in San Francisco, I could but attribute it, in contra-distinction, to the superior intelligence of my patient; who reasoned correctly when she thought that remedial promptings from her would but serve to render uncertain the natural deductions of my own judgment, and am truly thankful that my first serious case afforded me the benefit of such efficient co-operation on the part of my patient, and am convinced that she possesses that power of self-control over her passions and appetites that will afford her the assurance of freedom from relapse. Although, to her leading natural intelligence, acquired ability, and worldly experience, I feel myself chiefly indebted for the successful result, and would consider myself amply repaid by the knowledge gained through her skilled development of my innate powers of thoughtful deduction, I find myself; *perforce*, the possessor, in fee simple, of an elegant establishment that of itself attracts the class of patients most desirable, and the princessly gift is still more enhanced to my grateful pride, with the knowledge that she openly ascribes her recovery, from the much dreaded disease, to my treatment. In advocating with grateful expressions of praise her sense of my ability, I feel that she exceeds by far my just merits; but when I expostulated and expressed the fear that it would make us both appear ridiculous as of the mutual admiration class, she replied, "dear child, as you grow older in experience and your powers of analytical observation expand, you will become less sensitive with regard to your speech in conversation with the ordinary style of people that rank as first-class in society association; for as a general practice they neither think, reflect, nor remember, and if they attempt to repeat what they hear, are certain to make a muss of it,—and to their blunders, as with the Finns and Irish, I owe many of the hits that gained for me a reputation of humor and wit. The exceptions to this average are few and far between, as is merit with the well-patronized in your profession; for pretension in it, from the beginning, has held ruling supermacy, and I would much more readily employ an obscure well-educated person to treat me medically, than the usually successful professional parrot who has talked him or herself into popular society patronage; so you need have no fear of reaction from my over-gulling the gullible vanity of those who live to forget the useful experience of the past for the winged sensations produced by herding attractions."

Of course, I fell that I shall have your warmest sympathy and congratulations, not only for the successful treatment of my first case of this formidable disease, but in extreme pleasurable exaltation that the greatness of my recompense was not derived from publicly over-rating or insinuating the possession of extraordinary ability, or any other pretext for extortionate rating of my payment expectations; and I am sure that you cannot depreciate or regret more sincerely than I do my nnworthiness of the over-valuation placed upon the service rendered, when rated by the scale of worldly calculations, void of the consolatory expression of gratitude it tacitly expresses. * * * In the relation I have not specified the medicative means used in the different stages of the suppurative and healing process, as they were mainly of your suggestion and pre-paration, the variations were only those required to act in accordance with difference in constitutional and circumstantial impressions.

NOTE.—In the case of Mrs. P., referred to in the relation, the translation of the irritating cause for the development of the cancerous disease of osteo-sarcoma to the predisposed carious teeth and jaw, was more apparent than the description of the Russian example demonstrated. Mrs. P. was a Russian Jewess, born in Moscow, of Germo-Polish parentage, and had lived a migratory life; the first knowledge obtained of her habits and predispositions was while she was a resident of Panama, N. G., then a miss of eighteen; fifteen years after as a married woman she verified the prognosis of a latent cancerous tendency.

In her reckoned third pregnancy, the left inferior maxillary wisdom tooth, and anterior molars, became over-lapped with a fungus liver-hued growth, which was attended by a deep-seated throbbing pain in the jaw, to which her sensations referred it in contra distinction to tooth-ache; this extended to her left eye with neuralgic twitches. An examination showed a slight protrusion of the eye with contraction of the pupil, which remained immobile to the effect of belladonna, while the other seemed unduly sensitive to its influence. As the disease progressed the cause of this immobility became apparent from the induration and consequent contraction of the schlerotic membrane in counter compression to the protrusion from evident enlargement or tumefaction of the branch connections of the optic nerve with the posterior portions of the eye-ball, showing an anastomotic communication of the maxillary nerves with those of the eye, or more probable direct symptomatic translation of diseased irritation from the gravid uterus, as in cases of temporary myopia and in permanent, caused by excessive vagno-uterine excitement from masturbation and undue venery. Yet, in affecting a cure of the jaw osteosarcoma, the sight of the eye was improved and the induration and convexity abated, showing plainly that uncontrolled the disease would have extended to the eye.

In her case the disease was greatly aggravated by the continued use of the most indigestible substances, many of which could only be supposed to yield nutriment by a stretch of imagination; and it required the positive assurance of being left to her fate before she would submit to the alternative of a regulated diet. When the four teeth were extracted there was a copious flow of blood and fetid matter, which from coagulation rendered the application of caustic comparatively ineffective, and it was not until after the eleventh day that the suppurative process set in.

During the interval, from the extraction of the teeth to suppuration, the fungus growths were thrown out so rapidly that they over-reached the tongue and impeded breathing and threatened suffocation, so that portions had to be cut away, but with the advantage of allowing the caustic cup to compress and check the flow of blood, and by this course, reaction was established. At intervals, during the twelve months that it required to restore healthy action to the bones and membrane investments of the diseased parts, treatment was suspended to test the recuperative powers; but up to the time of her delivery of a still-born child, at full term, some act of imprudence would bring back all the local symptoms of her disease, that required renewed active treatment. The child, although well developed in the personals of Jewish idiosyncrasy, bore plainly the impress of its mother's disease. At the expiration of the twelfth month, from date of treatment, the patient was dismissed, with the warning that if she became pregnant again it would be almost certain to reproduce the cancerous predisposition, which would cause a consumption of the earthly constituents of the bones, and a lingering death, For five years she remained in comparatively good health. considering her habits, and then became pregnant again, but found a physician who aided her in procuring an abortion; after its accomplishment muscular power in locomotion gradually diminished, until it was with the greatest effort that she could succeed in crossing the room. In watching her movements, when attempting to walk, the want of stability in the long bones of her legs was very evident, and the wavering unsteadiness of her motions and lack of confidence in her voluntary powers, could be easily traced to the yielding bones from muscular contraction, they lacking necessary firmness to give direct elevation to the lower limbs in walking, until with a manifest daily decrease in ability she became utterly incapacitated, and when removed to the bed found it impossible to turn, unassisted, from side to side, or change her limbs or body with voluntary disposition from where they were placed. In this self-passive condition she was obliged to lay until a bed-sore formed over the sacral ridge and became cancerous and beyond curative reach, while the long bones of her lower limbs fractured one after another from the involuntary contraction of the muscles, cramped from long inaction and distention in one position, and when an easy death relieved her, the post-mortem dress-makers said "she could be folded together any way, as easy as though she had never a bone." But the bony texture of the jaw, the local seat of the first cancerous attack, retained its ossific firmness as perfect as the opposite ramus, although its cellular tissue had been exposed and partially recovered with a substitute for the cancellated structure, showing clearly that before the last source of irritation was engendered, her system was in the process of recuperation.—*The Dental Practitioner.*

